# Supplementary figures and images for: Deficiency in Lyst function leads to accumulation of secreted proteases and reduced retinal adhesion
Source: PLoS One. 2022 Mar 3;17(3):e0254469. doi: 10.1371/journal.pone.0254469 (PMC8893605; doi:10.1371/journal.pone.0254469)

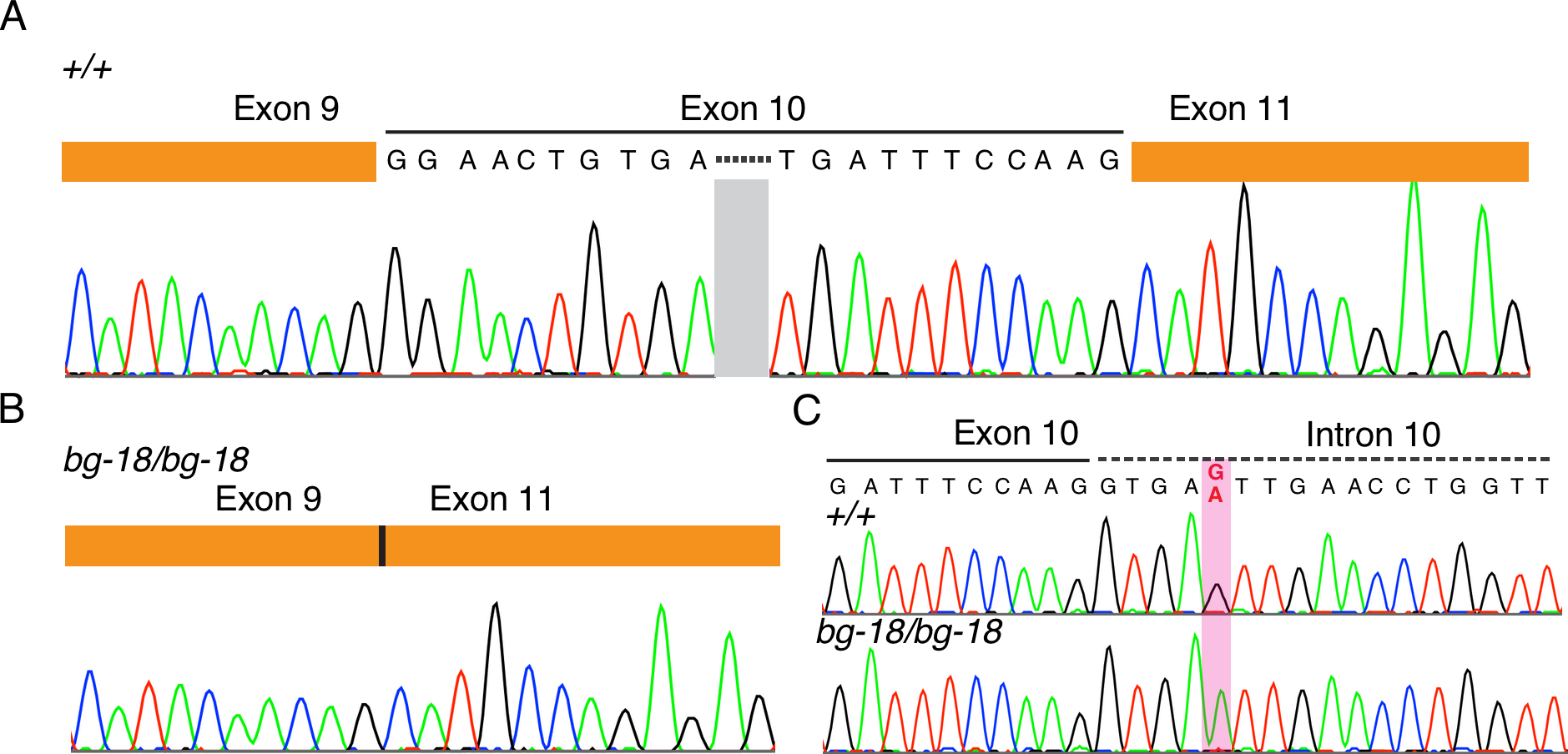

Supplement: S1 Fig — (A-B) cDNA sequences of the wild type (A) and mutant (B) around exon 10. Note the absence of exon 10 in mutant cDNA. (C) Genomic DNA sequences of the wild type (top) and mutant (bottom) around exon 10. The G to A transition is shaded in pink. (TIF) [file pone.0254469.s001.tif]

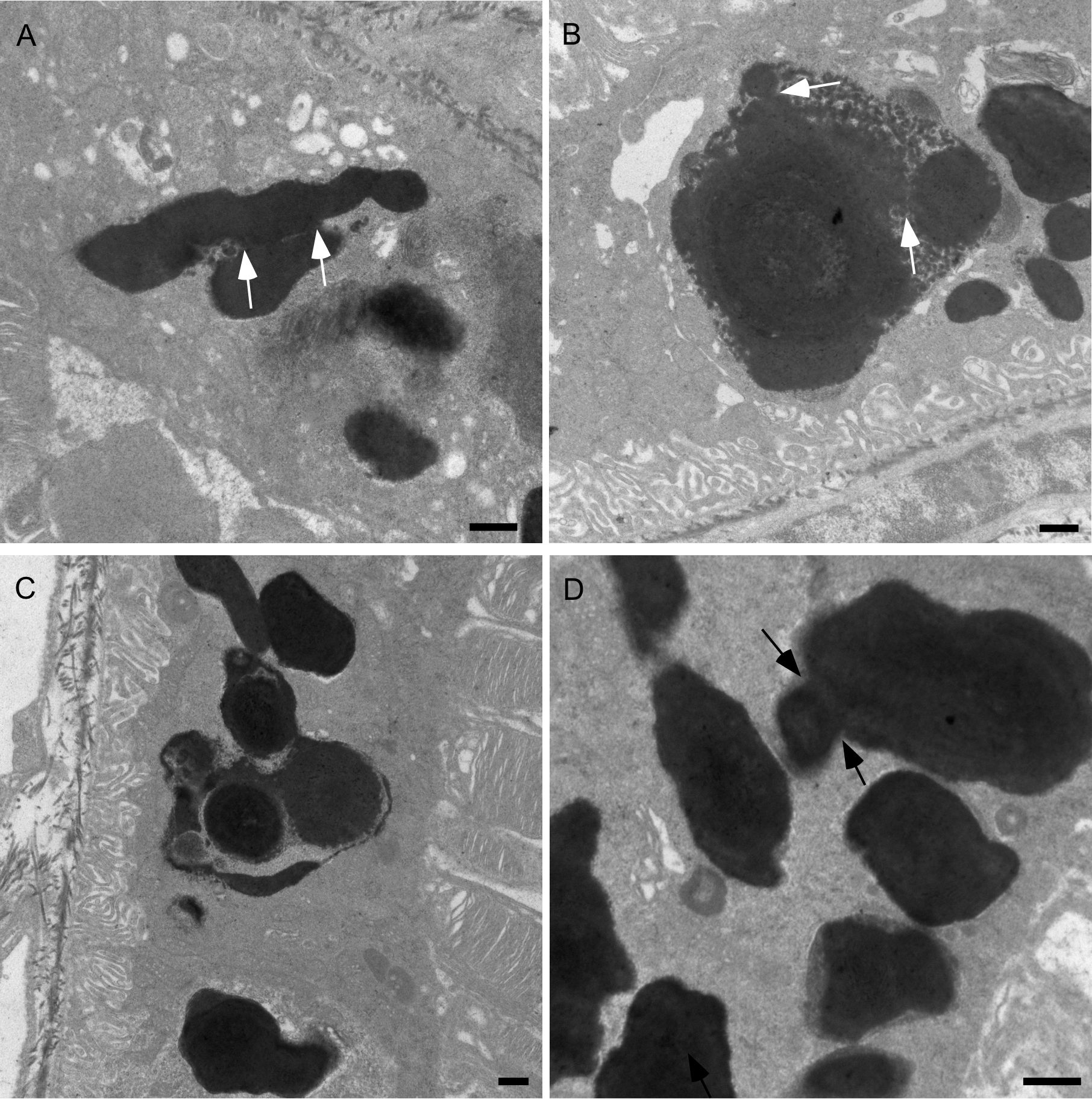

Supplement: S2 Fig — Arrows mark the locations of possible boundaries between melanosomes in the same cluster. Mice were 11-week-old upon euthanasia. Scale bar = 500 nm. (TIF) [file pone.0254469.s002.tif]

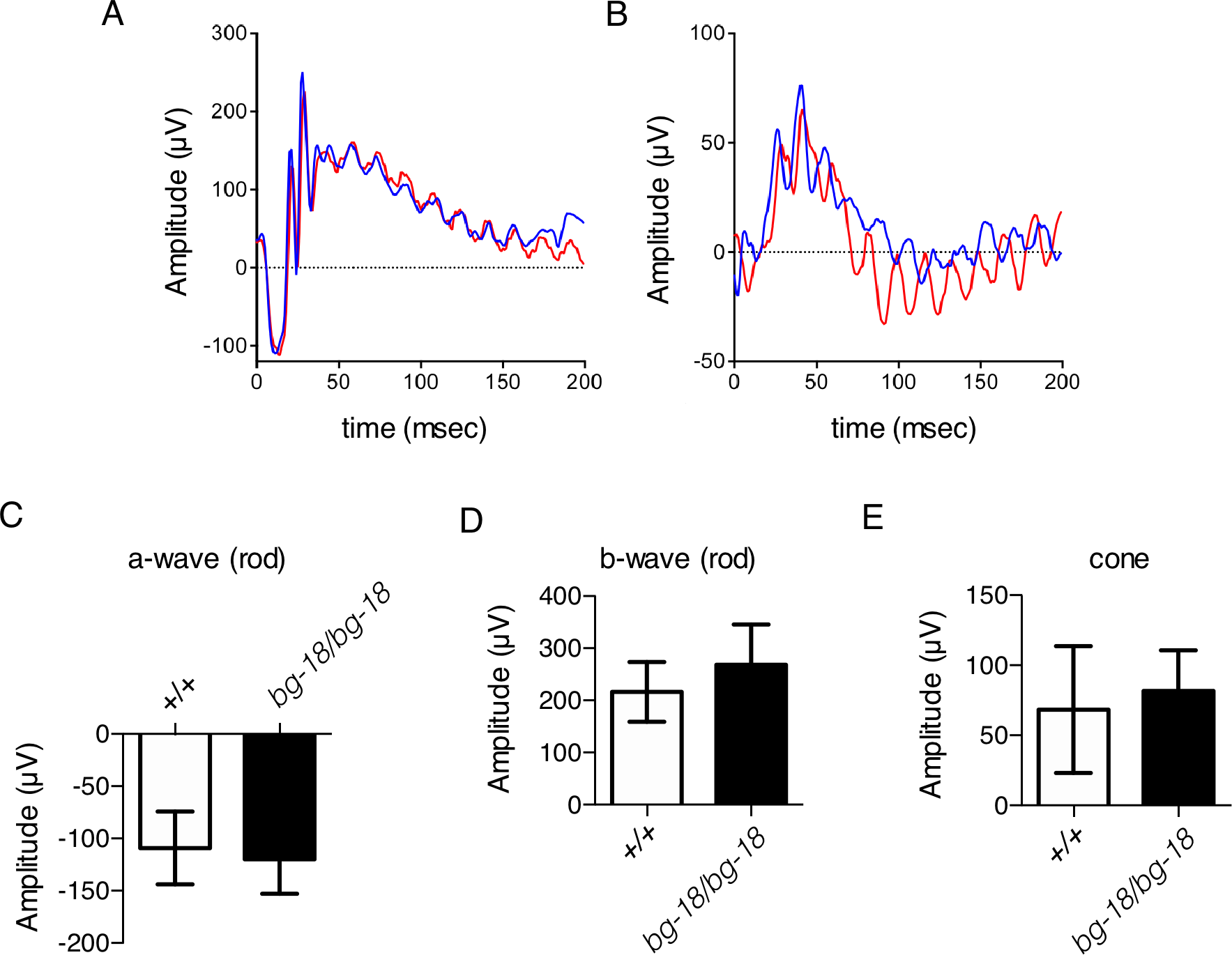

Supplement: S3 Fig — (A-B) Representative rod (A) and cone (B) ERG of the wild type (blue) and the mutant (red) eyes. (C-E) Quantified results of the a-wave (C) and b-wave (D) of rods, and the cone b-wave response (E). (TIF) [file pone.0254469.s003.tif]

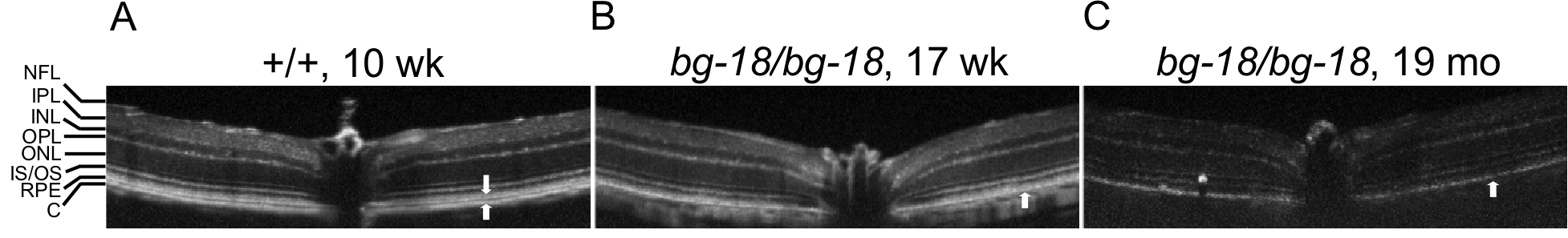

Supplement: S4 Fig — OCT images of the retina from 10-week-old wild type (+/+, A), 17-week-old mutant (B) and 19-month-old mutant mice (C). Retinal layers are labeled. NFL: Neurofilament layer; IPL: Inner plexiform layer; INL: Inner nuclear layer; OPL: Outer plexiform layer; ONL: Outer nuclear layer; IS/OS: Inner segment/outer segment; RPE: Retina pigment epithelium; C: Choroid. Note that there is only one hyper-reflective layer left at the location of RPE and choroid layers in the mutant retina (arrows). However, the overall thickness of the neuroretina is similar in the mutant eye compared with that in the wild type. (TIF) [file pone.0254469.s004.tif]

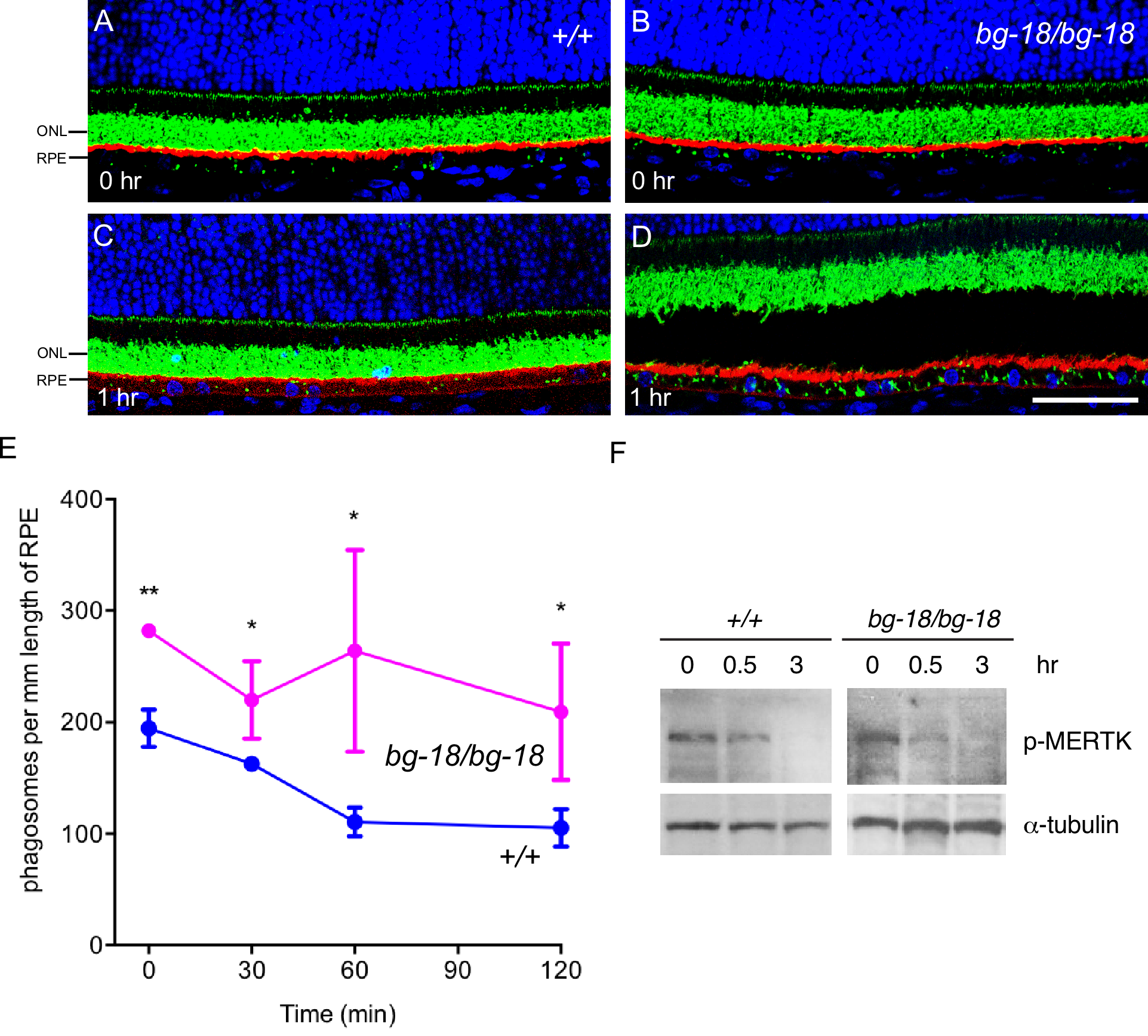

Supplement: S5 Fig — (A-D) Representative immunohistochemical images of wild type (A, C) and mutant (B, D) retina at 0 (A, B) and 1 (C, D) hour after onset of light, stained with antibodies against ZO-1 (red) and rhodopsin (green). Retinal layers are labeled. ONL: Outer nuclear layer; RPE: Retina pigment epithelium. Note that the sizes of phagosomes in the mutant RPE appear larger in general compared with the wild type RPE. Three mice of each genotype were used for each time-point. Scale bar = 50 μm. (E) Quantified results showing the number of phagosomes in the wild type (blue) and mutant (pink) RPE at 0, 30, 60 and 120 minutes after light onset. Phagosomes from 2 equally-sized areas of each retina section were counted in samples from 3 wild type and 3 mutant mice. *: P < 0.05; **: P < 0.01. Two-tailed t-test. (F) Phospho-MERTK level was reduced after the onset of light in both the wild type and the mutant retina. Western blot analysis showing p-MERTK level at 0, 0.5 and 3 hour after light onset in the wild type (+/+) and the mutant (bg-18/bg-18) eyecups. Alpha-tubulin was used as the loading control. All mice used in this figure were 5-week-old at time of euthanasia. (TIF) [file pone.0254469.s005.tif]

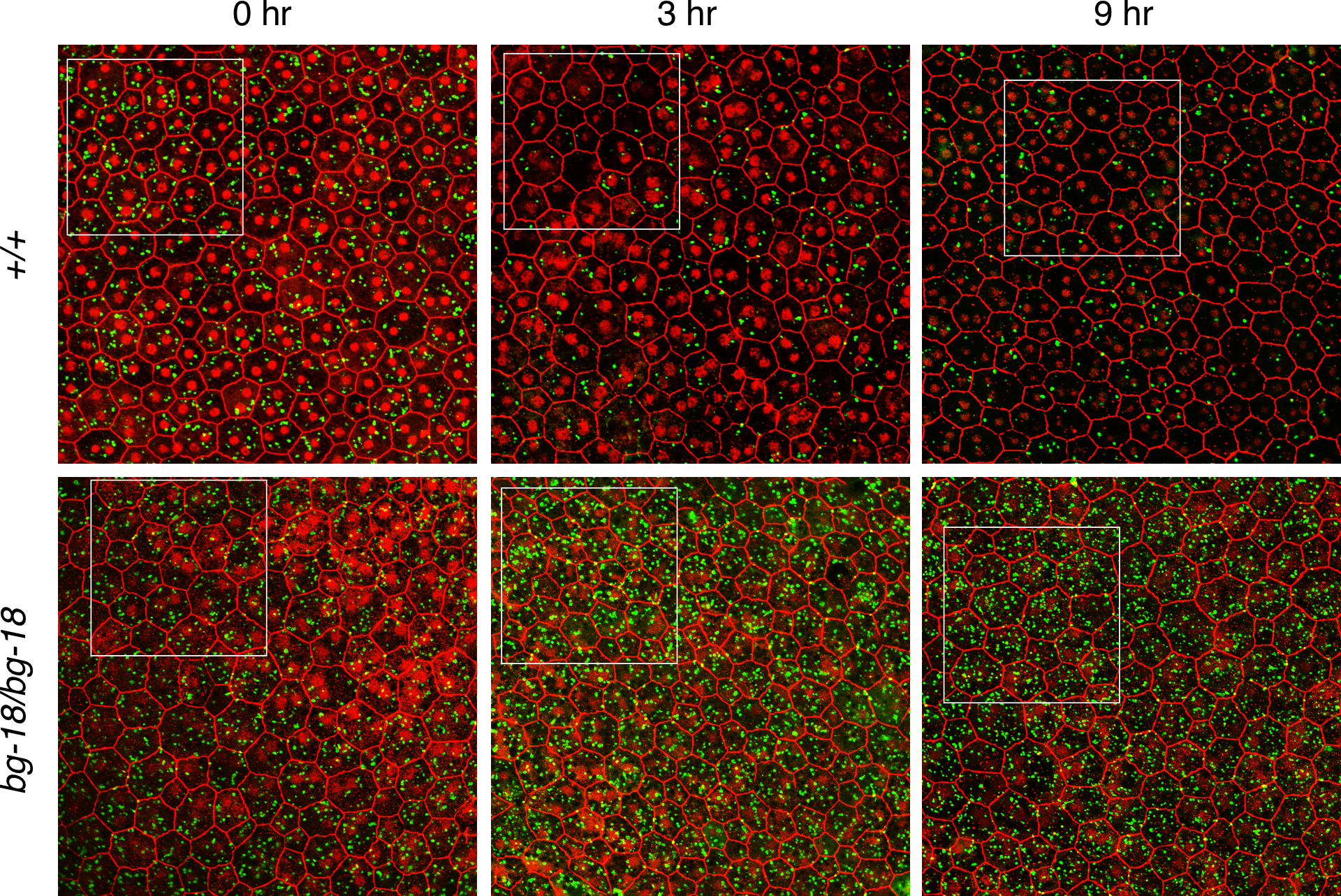

Supplement: S6 Fig — RPE from 5-week-old wild type (A-C) and bg-18 mutant (D-F) mice was incubated with antibodies against ZO-1 (red) and rhodopsin (green), after dissection at 0 (A, D), 3 (B, E), and 9 (C, F) hours after the onset of light at 6 AM. 3 mice of each genotype were examined for each time-point. The images show single confocal z-sections. Areas circled with squares are also enlarged and shown in Fig 2. (TIF) [file pone.0254469.s006.tif]

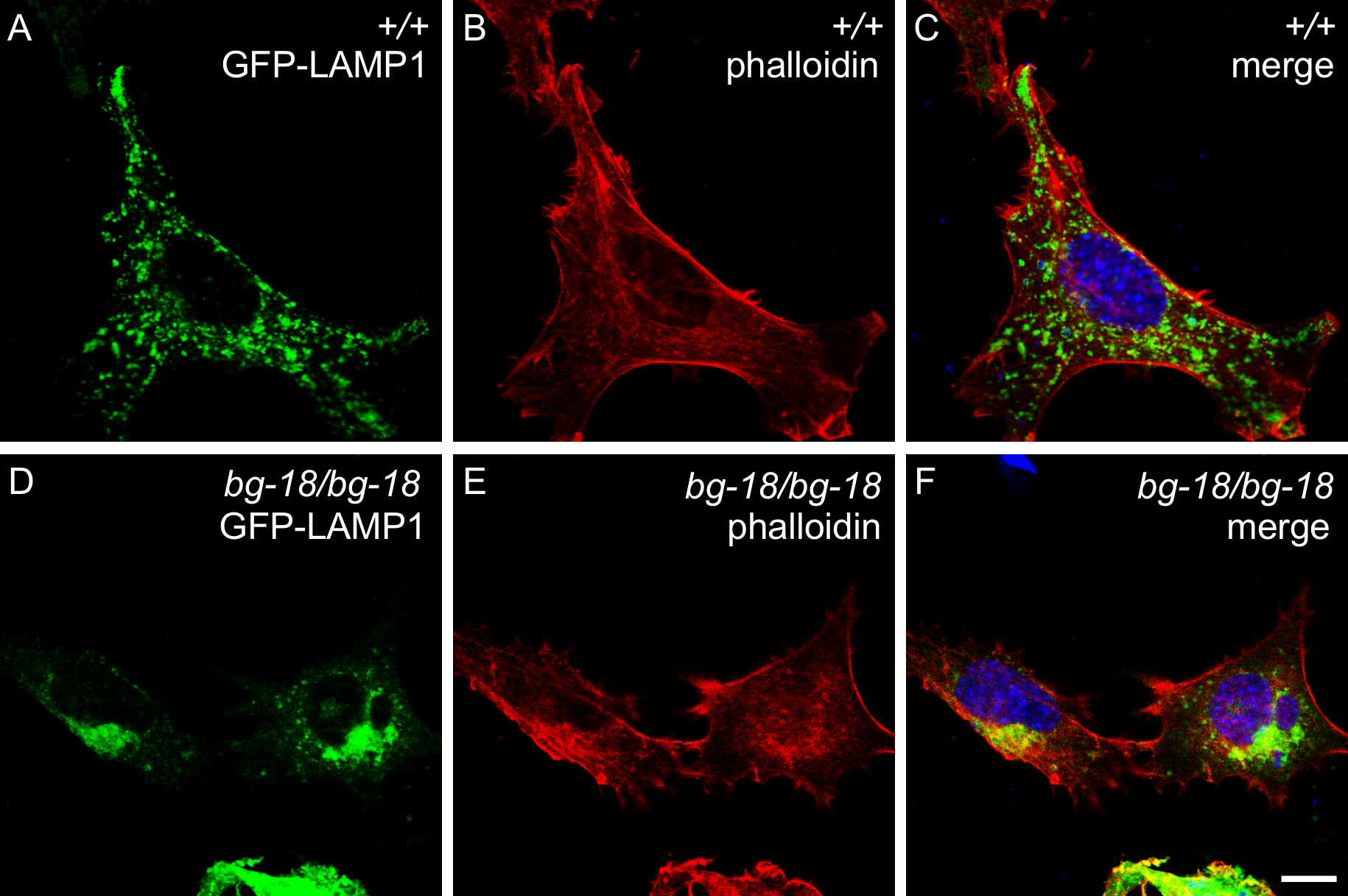

Supplement: S7 Fig — Wild type (A-C) and mutant (D-F) MEFs were transiently transfected with a plasmid expressing GFP-LAMP1 (A, D), and counterstained with phalloidin (B, E). The merged images are shown in (C, F). Scale bar = 10 μm. (TIF) [file pone.0254469.s007.tif]

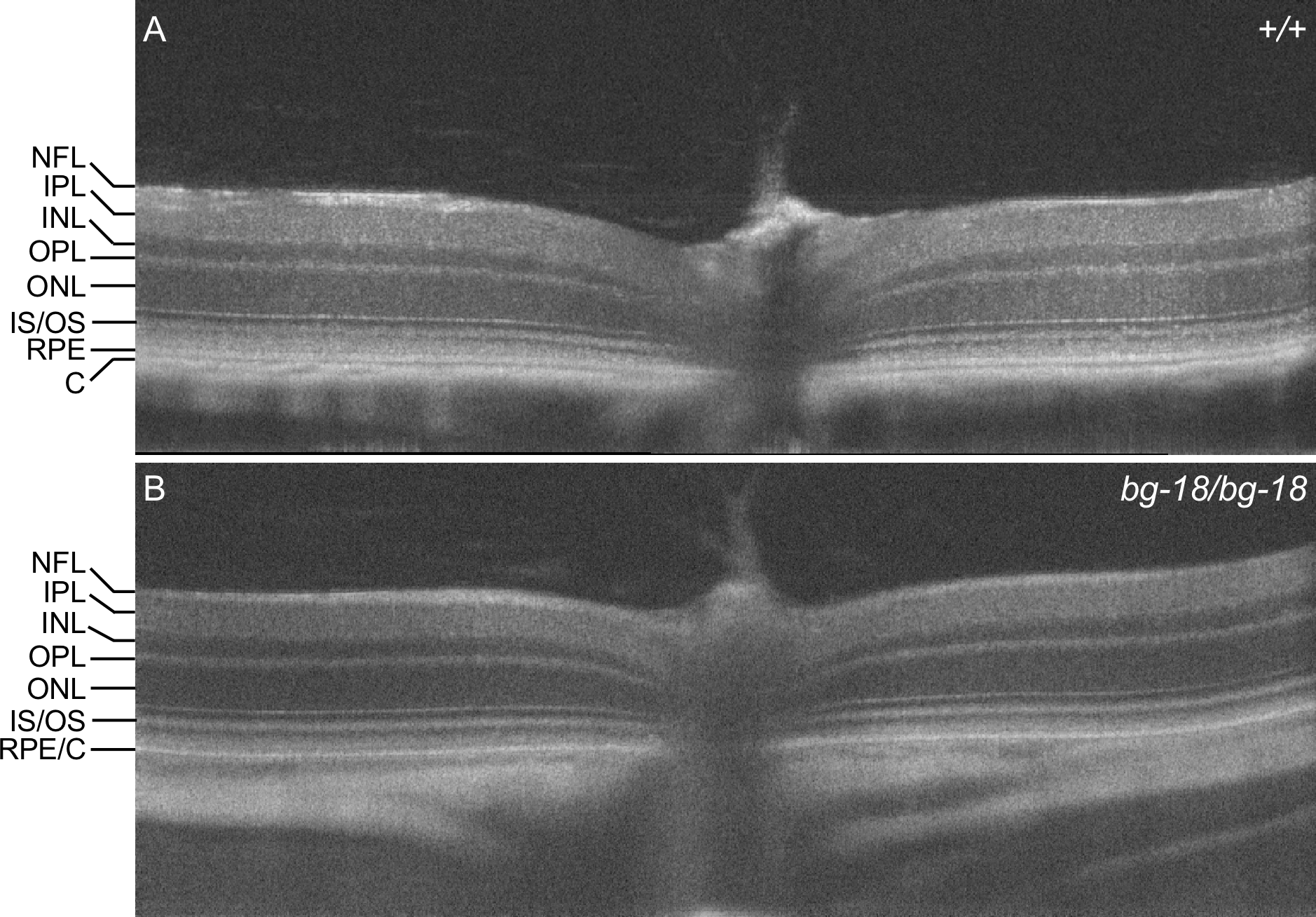

Supplement: S8 Fig — Representative OCT sections from 2-month-old wild type (A) and mutant (B) retina are shown. Each image is the average of a stack of 50 scans. Retinal layers are labeled as in S4 Fig. Note the photoreceptor outer segment (OS) layer is not detached from underlying layers. In addition, there are two hyper-reflective layers corresponding to RPE and choroid, respectively, in the wild type retina, but only one layer is observed at the same location in the mutant retina. (TIF) [file pone.0254469.s008.tif]

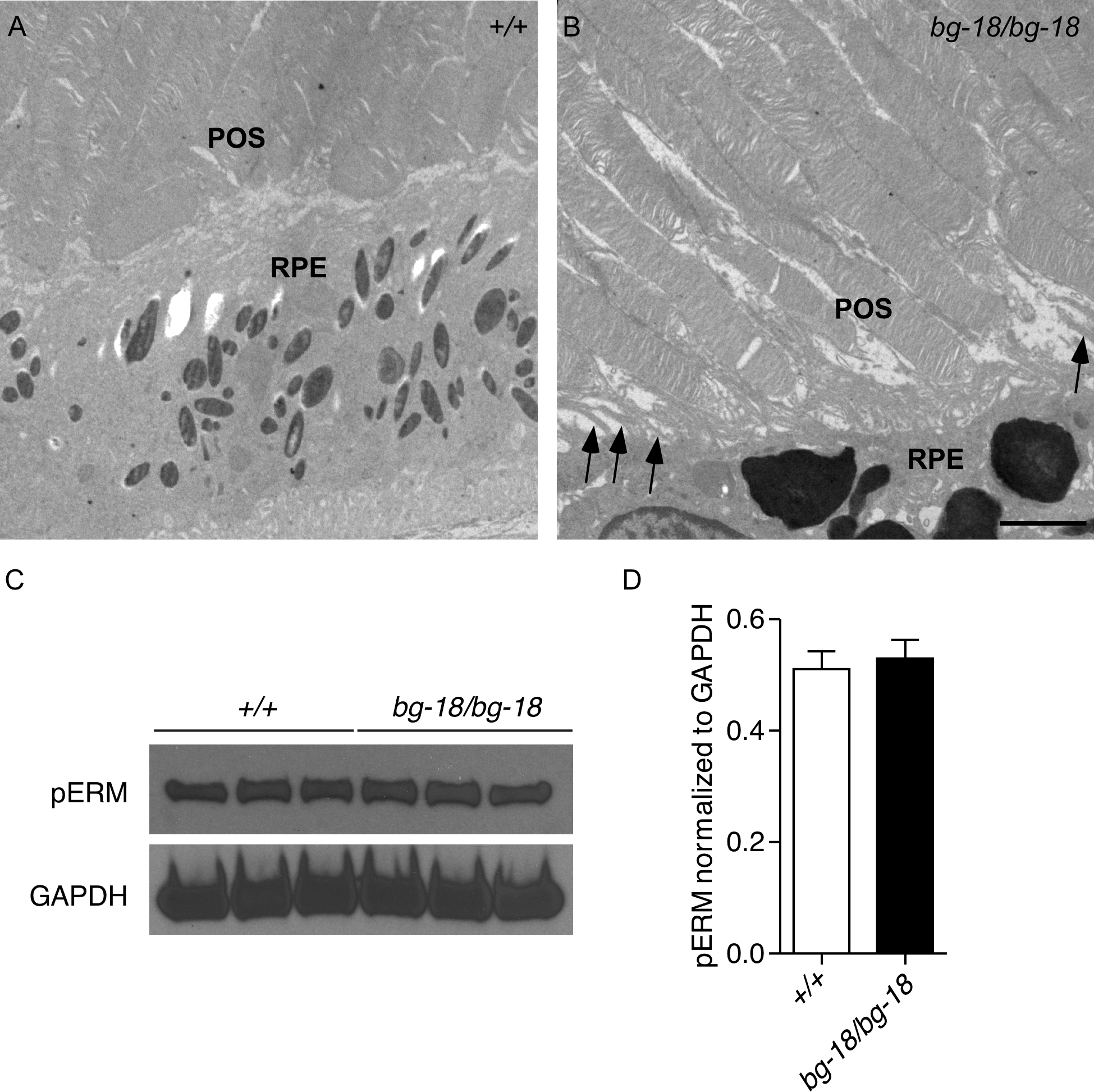

Supplement: S9 Fig — (A-B) Transmission electronic micrographs of 11-week-old wild type (A) and mutant (B) retina showing the photoreceptor outer segments (POS) and RPE. Several RPE microvilli in the mutant retina are marked with arrows. Scale bar = 2 μm. (C-D) Western blot analysis (C) showing phospho-ERM in 5-week-old wild type (+/+) and homozygous mutant eye cups. The results were quantified in (D). Note the similar pERM levels after normalizing to the internal loading control GAPDH. (TIF) [file pone.0254469.s009.tif]

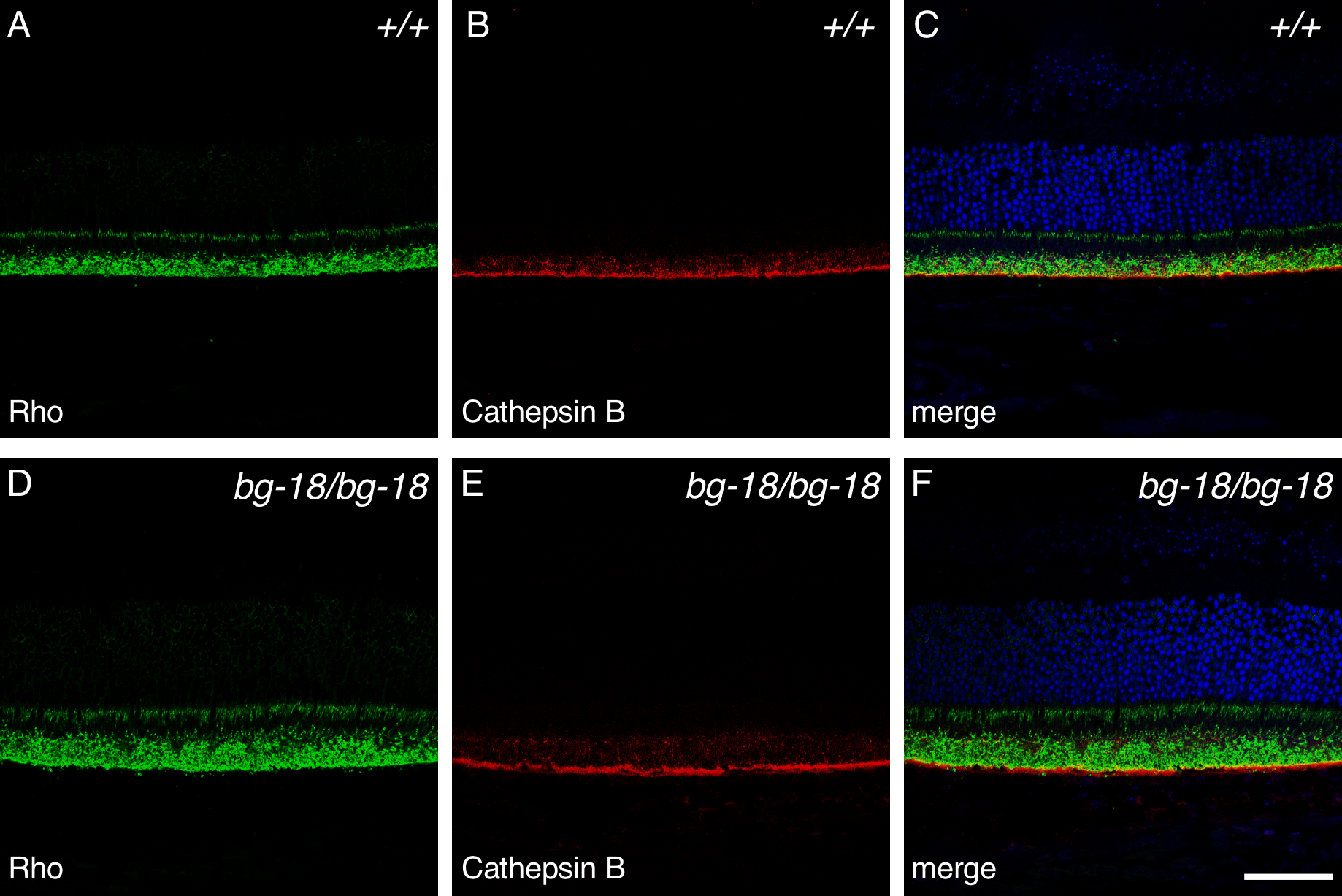

Supplement: S10 Fig — Wild type (A-C) and mutant (D-F) retina sections from 4-week-old mice were subjected to immunostaining with rhodopsin (A, D) and cathepsin B (B, E). The merged results (C, F) show the localization of rhodopsin and cathepsin B layers in proximity. Retinal layers are labeled as in Fig 4. Scale bar = 50 μm. (TIF) [file pone.0254469.s010.tif]
