# Supplementary material for: Deficiency in Lyst function leads to accumulation of secreted proteases and reduced retinal adhesion
Source: PLoS One. 2022 Mar 3;17(3):e0254469. doi: 10.1371/journal.pone.0254469 (PMC8893605; doi:10.1371/journal.pone.0254469)
Supplement: S1 Raw images — (PDF) [file pone.0254469.s011.pdf]

Fig. 2H

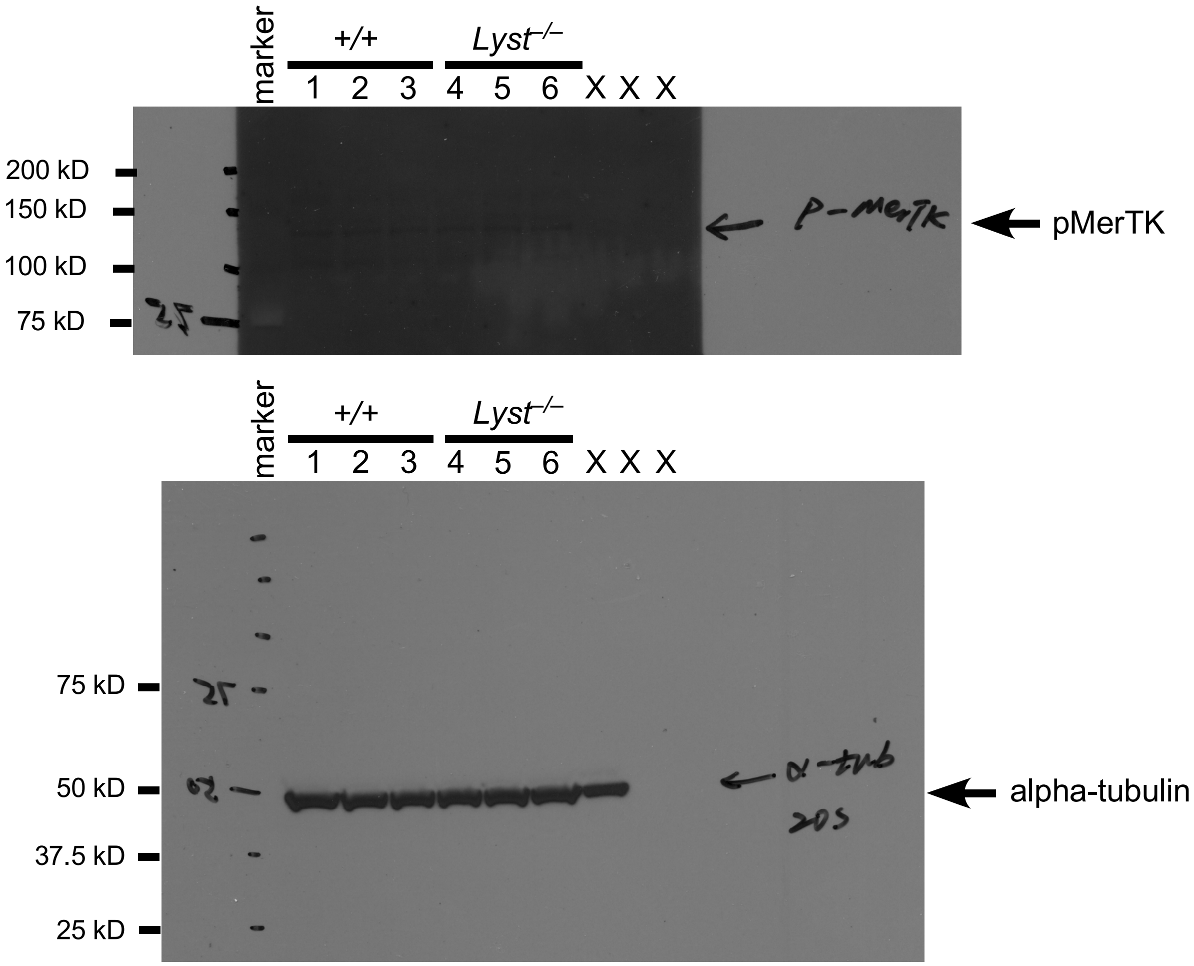

Because of the high background of pMerTK western, blots could not be stripped. Separate gels and blots were used. Blots were exposed to X-ray films and scanned with a flat-bed scanner. No image enhancement was done here.

Fig. 4C neuroretina

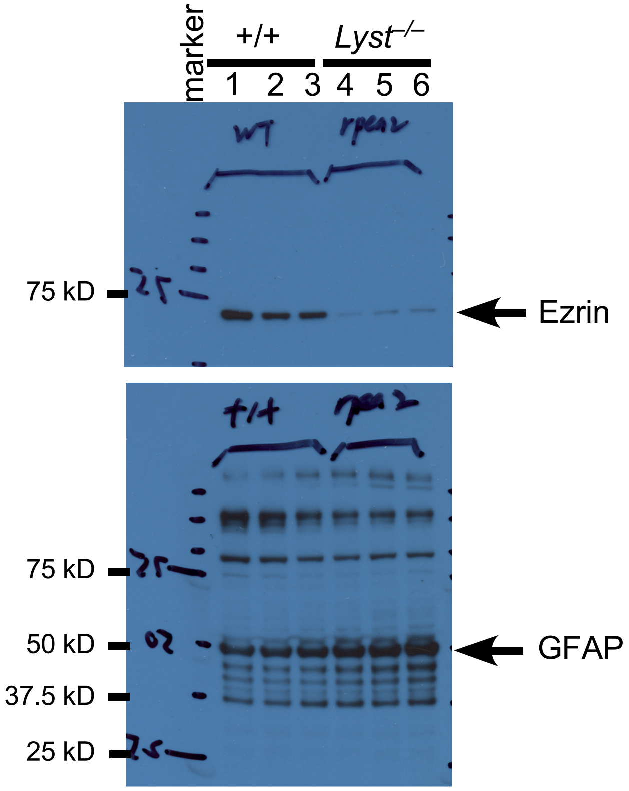

Stripped and reprobed

Fig. 4E Eye cup

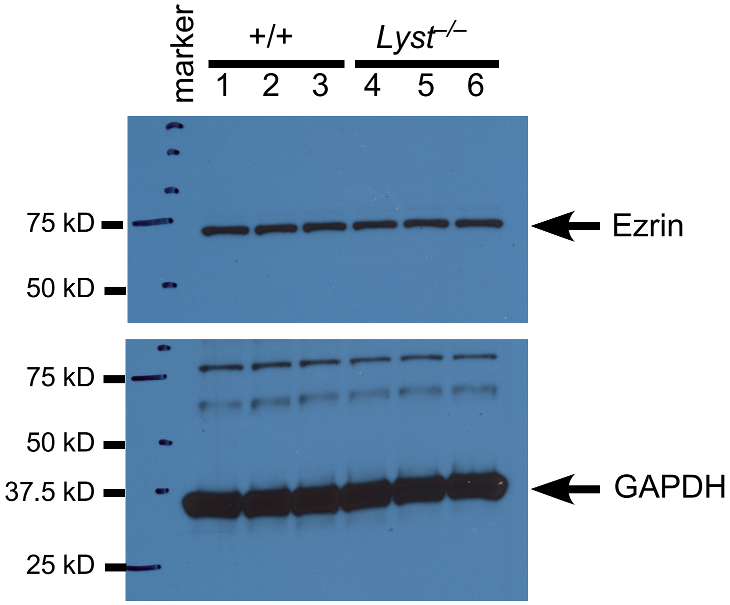

Stripped and re-probed

Blots were exposed to X-ray films and scanned with a flat-bed scanner.
No image enhancement was done here.

Fig. 5E

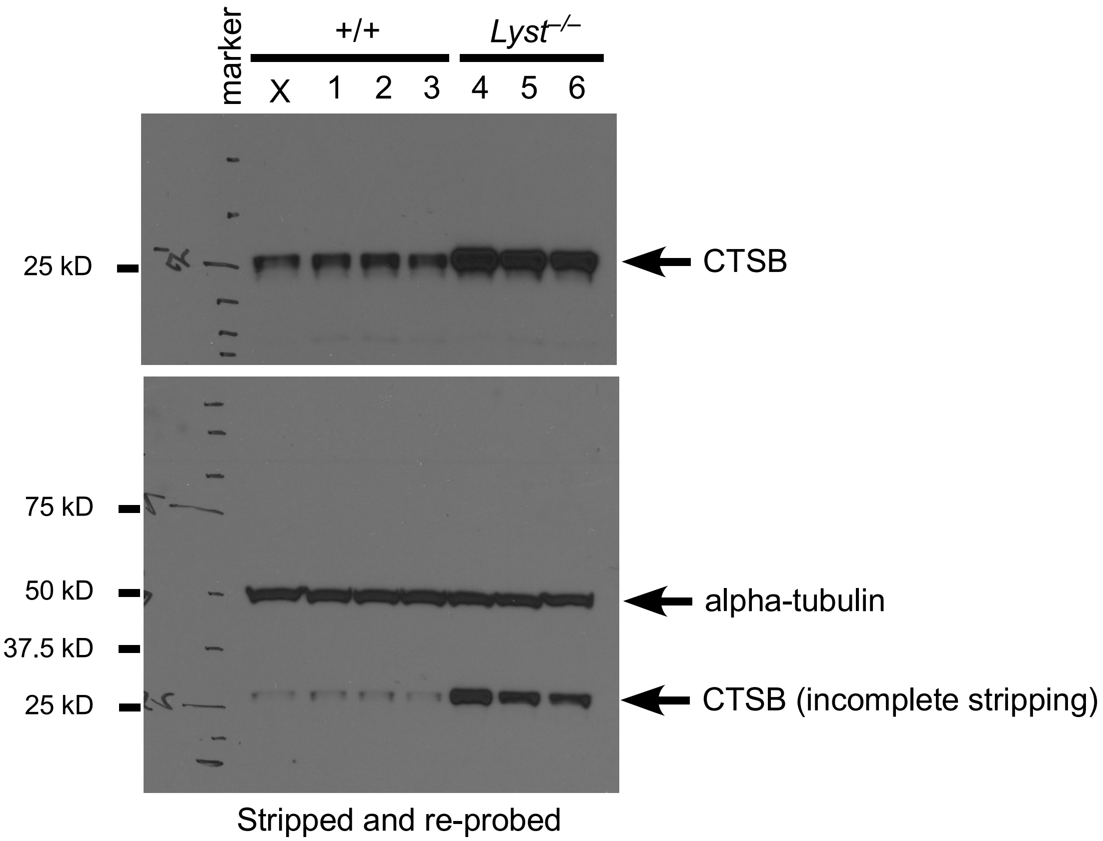

Blots were exposed to X-ray films and scanned with a flat-bed scanner.
No image enhancement was done here.

Fig. 6A

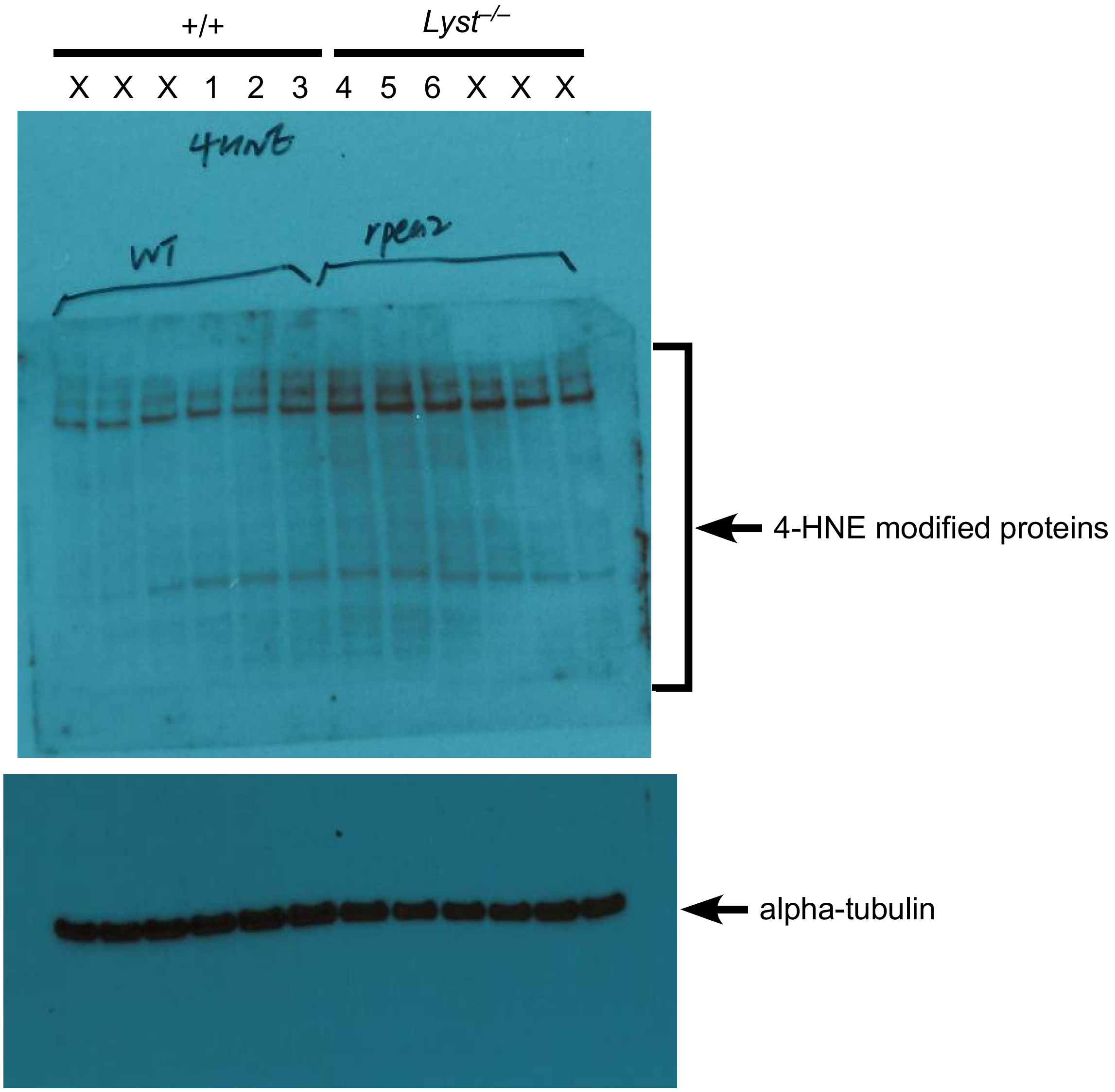

We used 12 well gel to get better resolution and we would like to have 6 samples for each genotype to avoid the effect of sample variation since we uses an antibody against a modification but not a particular protein. We did not include markers because of this. However, we gauged the sizes of the major bands by blots from preliminary experiments that we used to test different denaturation conditions.
Blots were exposed to X-ray films and scanned with a flat-bed scanner.
No image enhancement was done here.

Fig. S5

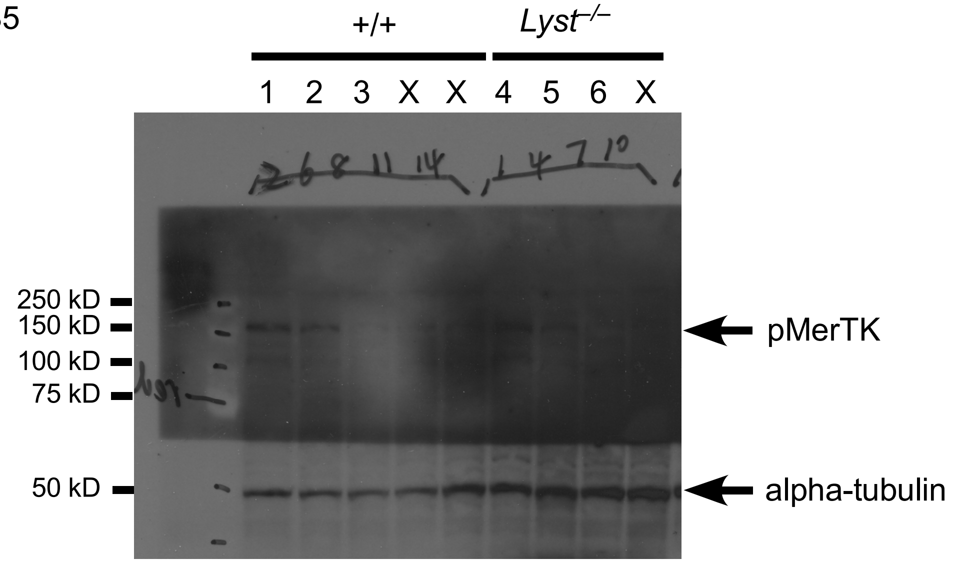

Blots were exposed to X-ray films and scanned with a flat-bed scanner.
2 blots were exposed to the same film simultaneously
No image enhancement was done here.

Fig. S9

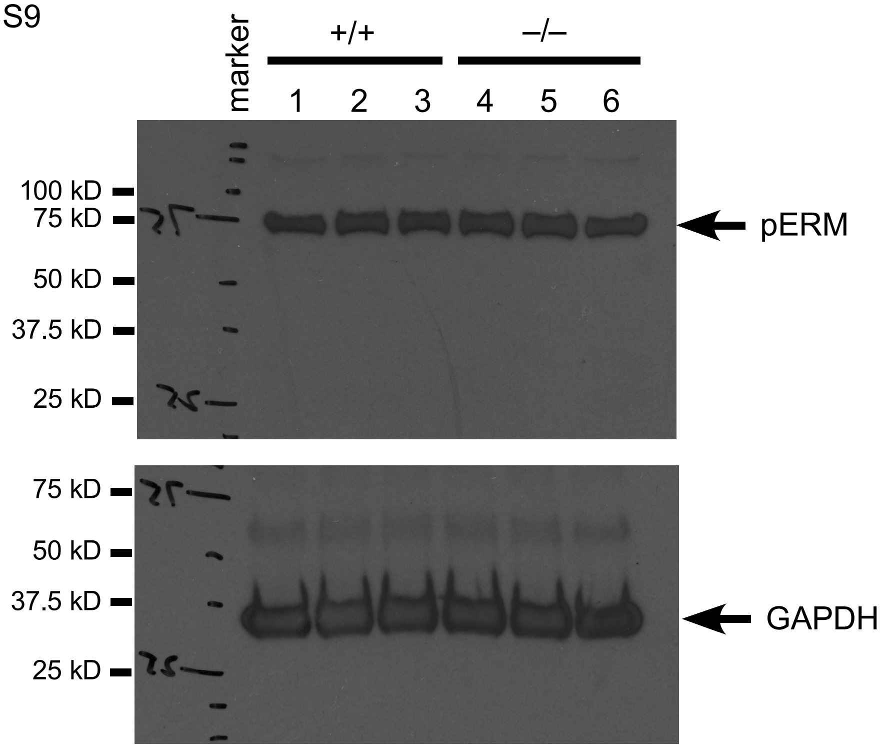

Blots were exposed to X-ray films and scanned with a flat-bed scanner.
No image enhancement was done here.
