# Supplementary material for: Deficiency in Lyst function leads to accumulation of secreted proteases and reduced retinal adhesion
Source: PLoS One. 2022 Mar 3;17(3):e0254469. doi: 10.1371/journal.pone.0254469 (PMC8893605; doi:10.1371/journal.pone.0254469)
Supplement: S1 File — Additional details of methods used for this manuscript and supporting information. (PDF) [file pone.0254469.s012.pdf]

Supporting Text

Material and Methods

Genome mapping and identification of the *Lyst*$^{bg\text{-}18J}$ mutation in the genome

The *nm2144* mutation, which arose spontaneously as a recessive mutation on the C57BLKS/J background in the Mouse Mutant Resource colony, was backcrossed to C57BL/6J for more than 10 generations to create a congenic strain, before we performed linkage analysis using the fundus phenotype. Simple sequence length polymorphic (SSLP) markers in the candidate region were tested. Based on the similarity of the fundus images of *beige* (*Lyst*$^{beige\text{-}J}$, JR000629, The Jackson Laboratory) and *nm2144* mutant mice, *D13Mit17*, an SSLP marker close to the *Lyst* locus, was used to test cosegregation of the phenotype and the marker, using 23 F2 mice from the intercross between the *nm2144* mutant and C3A.BLiA-Pde6b+/J. We found that the fundus phenotype of *nm2144* mutants co-segregated with the polymorphic sequence-tagged site (STS) marker *D13Mit17*.

To confirm the linkage analysis result, an allelism test was conducted by crossing the *Lyst*^{bg} mutant and the *nm2144* strain. The resulting progeny were compound heterozygotes for *nm2144* and *beige-J.* Their fundus phenotype and the coat color were assessed. These compound heterozygous mice exhibited the same fundus phenotype as *nm2144* or *beige-J* homozygotes, indicating that *nm2144* and *beige-J* are allelic.

For mutational analysis, fragments of *Lyst* cDNA were amplified by RT-PCR and

sequenced. RT-PCR products amplified from eyecups using the oligo primers LystcDNAseq1F (5'-ACGGGGAAATAATATACCCTGA-3') and LystcDNAseq1R (5'-GTCAGACACTGTGAAGGGCTCA-3'), which binds to exon 9 and exon 11, respectively, were sequenced, which led to the identification of the missing exon 10 in *nm2144* mutant cDNA (Fig. S1A, B). To identify the mutation leading to the exon 10 deletion, the primers - LystGenoseq1F (5'-CGCAGACACACACACTCTCTCT-3') and LystGenoseq1R (5'-AGCATGAGGAACAAACAGGACG-3') were used to amplify genomic DNA from WT and mutant mice, followed by sequencing. Genomic DNA sequences adjacent to exon 10 of the *Lyst* gene showed a guanine to adenine nucleotide transition near the intron 10 splice donor site, leading to the skipping of exon 10 and a frameshift after exon 9 that results in a premature stop codon in exon 11 (Fig. S1). Therefore, we named this mutation, *beige* (*bg*)-18J.

Quantitative real-time PCR

Oligonucleotide primers for qRT-PCR were designed using the Benchling server (https://benchling.com). The forward primer, mLystQ1F (5'-TTTGATGCCCTGCTTCGAGT-3') and reverse primer, mLystQ1R (5'-GAAAAATCTCCGGGCTGGGA-3') were designed to bind to exon 7 and exon 8 of the *Lyst* transcript, respectively. The expected size of the RT-PCR product was 122 bp. Another set of primers, mLystQ2F (5'-ATCCAAGTGTGGGCTGATCC-3') and mLystQ2R (5'-TCCTGTGATTCCACCTGTGC-3') that bound to exon 13 and exon 14 of the *Lyst* transcript, respectively, were also used. For internal control, the GAPDH sequence was

amplified using primers, GAPDH-QF (5′-CTTTGTCAAGCTCATTTCCTGG-3′) and GAPDH-QR (5′-TCTTGCTCAGTGTCCTTGC-3′). In addition, a *Ctsb* (Cathepsin B) cDNA fragment was amplified with the primers, Ctsb-F (5'-GGTACTTAGGAGTGCACGGGAG-3') and Ctsb-R (5'-AAGGACCACCACATCCTGGATG-3'). A *Ctsl* (Cathepsin L) cDNA fragment was amplified with the primers, Ctsl-F (5'-CTTTTGGCTGTCCYCYGCTTGG-3') and Ctsl-R (5'-TCTCCCATATCGCTCTCCTCCA-3'). A *Ctss* (Cathepsin S) cDNA fragment was amplified with the primers, Ctss-F (5'-CTCTGTGGCAATGGAGCAACTG-3') and Ctss-R (5'-CCAGATGAGACGCCGTACTTCT-3'). An *Mmp3* cDNA fragment was amplified with the primers, Mmp3-F (5'-TGTGCTCATCCTACCCATTGCA-3') and Mmp3-R (5'-TGTCATCTCCAACCCGAGGAAC-3').

For each reaction, 1 μL cDNA and 5 pmol primers were used. The PCR program contained a melting curve step at the end of the assay. The PCR products were resolved on a 3% Metaphor agarose gel to confirm the correct product size. The real-time PCR results were analyzed initially with the Bio-Rad CFX Manager software package. The three technical replicates were first averaged and results for each biological sample were analyzed. The relative expression data was exported and further analyzed in Prism 6 (GraphPad, San Diego, CA, USA) to calculate the mean and standard deviation of biological replicates. Unpaired *t*-test was performed to determine if differences between WT and mutant mice was statistically significant.
